# Label-free imaging of M1 and M2 macrophage phenotypes in the human dermis in vivo using two-photon excited FLIM

**DOI:** 10.7554/eLife.72819

**Published:** 2022-10-06

**Authors:** Marius Kröger, Jörg Scheffel, Evgeny A Shirshin, Johannes Schleusener, Martina C Meinke, Jürgen Lademann, Marcus Maurer, Maxim E Darvin

**Affiliations:** 1 Charité – Universitätsmedizin Berlin, corporate member of Freie Universität Berlin, Humboldt- Universität zu Berlin, and Berlin Institute of Health, Department of Dermatology, Venerology and Allergology Berlin Germany; 2 Lomonosov Moscow State University, Faculty of Physics Moscow Russian Federation; University of Oxford United Kingdom; CNRS LPENS France

**Keywords:** skin, dermis, immune cells, phagocytosis, confocal microscopy, non-invasive, Human

## Abstract

Macrophages (ΜΦs) are important immune effector cells that promote (M1 ΜΦs) or inhibit (M2 ΜΦs) inflammation and are involved in numerous physiological and pathogenic immune responses. Their precise role and relevance, however, are not fully understood for lack of noninvasive quantification methods. Here, we show that two-photon excited fluorescence lifetime imaging (TPE-FLIM), a label-free noninvasive method, can visualize ΜΦs in the human dermis in vivo. We demonstrate in vitro that human dermal ΜΦs exhibit specific TPE-FLIM properties that distinguish them from the main components of the extracellular matrix and other dermal cells. We visualized ΜΦs, their phenotypes and phagocytosis in the skin of healthy individuals in vivo using TPE-FLIM. Additionally, machine learning identified M1 and M2 MФs with a sensitivity of 0.88±0.04 and 0.82±0.03 and a specificity of 0.89±0.03 and 0.90±0.03, respectively. In clinical research, TPE-FLIM can advance the understanding of the role of MФs in health and disease.

## Introduction

**Figure 1.**
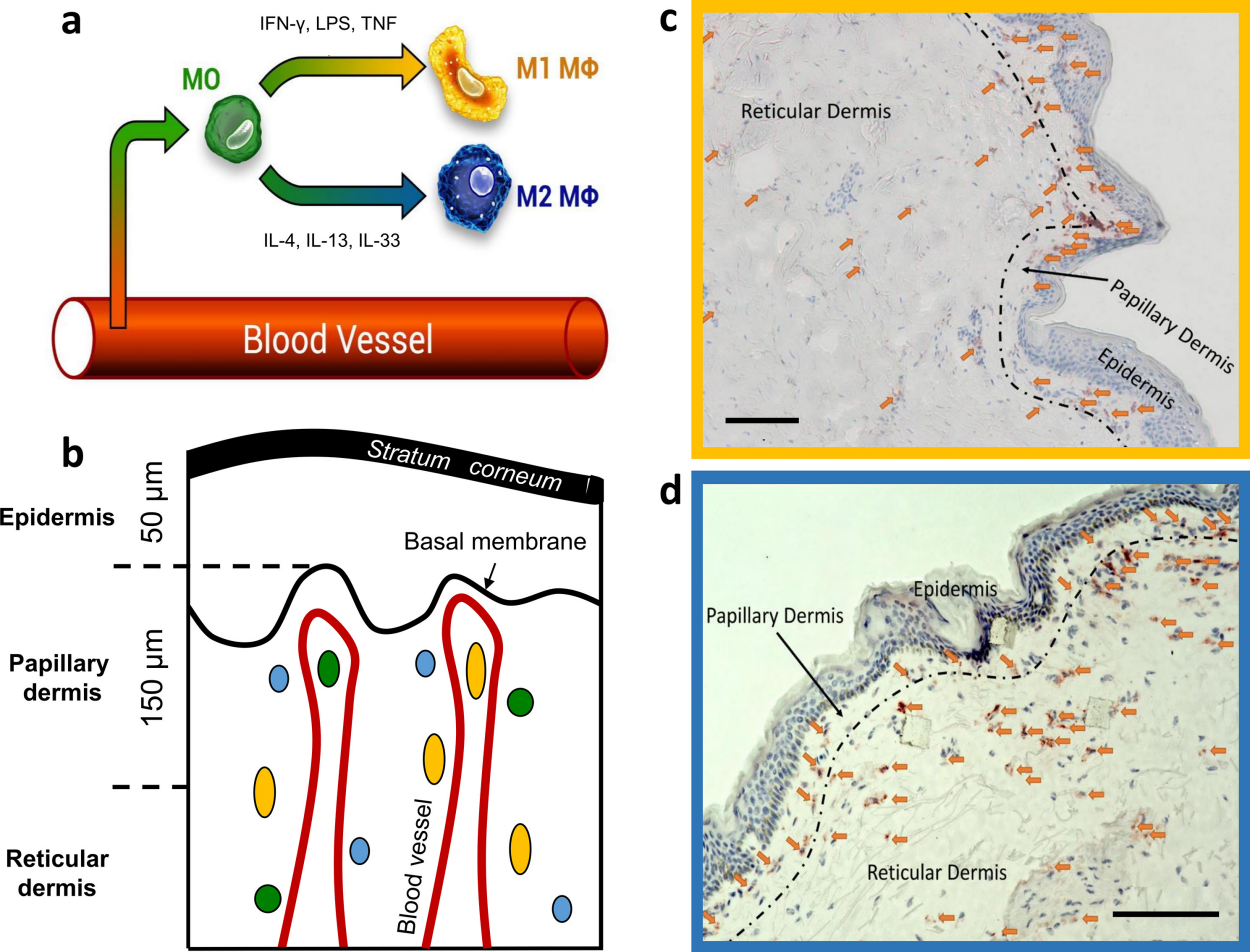
Dermal monocyte skin infiltration and CD68 stained M1 and CD163 stained M2 ΜΦs distribution in excised human skin. Schematic illustration of monocyte (MO) (green) infiltration into tissues and macrophage (ΜΦ)-polarization into M1 ΜΦs (yellow) via IFN-γ, LPS, and TNF and M2 ΜΦs (blue) via IL-4, IL-13, and IL-33 (**a**). Schematic of skin with exemplary locations of monocytes (green), M1 ΜΦs (yellow), and M2 ΜΦs (blue) (**b**). Density of M1 ΜΦs (marked with arrows) stained with CD68 (**c**) and M2 ΜΦs (marked with arrows) stained with CD163 (**d**) in 10 µm thick cryo-section. Scale bar: 100 µm. The terms M1 and M2 ΜΦs are simplistic, as many signals modulate MФ functions, resulting in a spectrum between the M1 and M2 MФ phenotypes.

Macrophages (ΜΦs) are important immune effector cells in organs and tissues that act as border junctions to environments such as the gut, the airways, and the skin (Elhelu, 1983). Skin ΜΦs (Dong et al., 2016; Estandarte et al., 2016; Ryter, 1985) originate from circulating monocytes (Geissmann et al., 2010; Gordon and Taylor, 2005) via the same infiltration route into the dermis as monocyte-derived dendritic cells (Schmid and Harris, 2014; Figure 1a) and are mainly located in the papillary and reticular dermis in close proximity to blood vessels (Weber-Matthiesen and Sterry, 1990; Figure 1b–d). It has been known for more than 30 years that skin ΜΦs are abundant and heterogeneous, based on their morphology, localization, and staining properties (Weber-Matthiesen and Sterry, 1990). More recently, skin ΜΦs have been classified based on their function, and they fall into two phenotypes referred to as inflammation-promoting M1-polarised ΜΦs (classically activated) and anti-inflammatory M2-polarised ΜΦs (alternatively activated) (Arango Duque and Descoteaux, 2014; Figure 1a). M1 ΜΦs are activated by viral and bacterial infection (Benoit et al., 2008; Ferrer et al., 2019; Malmgaard et al., 2004), interferon-γ, lipopolysaccharide (LPS), and tumor necrosis factor (TNF), which is known as the classical activation pathway (Li and Liu, 2018). M2 ΜΦs are alternatively activated in response to IL-4, IL-13, and IL-33 (Furukawa et al., 2017; Sica and Mantovani, 2012). Recently, this paradigm was questioned, as a manifold of cytokines, biomarkers, and activators are involved in MФs functioning, resulting in a continuum of states between the M1 and M2 phenotypes (Mendoza-Coronel and Ortega, 2017; Murray et al., 2014). Furthermore, different markers like CXCL10 for M1 ΜΦs and CCL17 for M2 ΜΦs can have the function to attract t-cells. Macrophages in disease, cancer, or obesity can switch function from wound healing to inflammatory ΜΦs given the right signals and microenvironment (Mosser and Edwards, 2008). However, for simplicity, the terms M1 and M2 ΜΦs are used here with the activators in brackets, where applicable.

Skin M1 ΜΦs are held to contribute to dermal innate immunity and homeostasis. This is supported by reports that M1 ΜΦs can phagocyte objects up to 20 µm in size (Morhenn et al., 2002), promote skin inflammatory and immune responses (Remmerie and Scott, 2018; Theret et al., 2019; Yanez et al., 2017), and produce nitric oxide and other reactive oxygen species (ROS) (Forman and Torres, 2001; Rendra et al., 2019). Skin M2 ΜΦs, on the other hand, are thought to promote dermal repair, healing, and regeneration, for example, by contributing to the formation of the extracellular matrix (ECM; Ploeger et al., 2013).

The precise role of skin ΜΦs and their M1 and M2 phenotypes in health and disease remain to be elucidated. In skin diseases, such as melanoma (Bardi et al., 2018), systemic sclerosis (Trombetta et al., 2018), Lupus (Chong et al., 2015), and LPS tolerance (O’Carroll et al., 2014), polarization of ΜΦs leading to mixed M1 and M2 phenotypes can be observed. It is not known whether and in what density mixed ΜΦ phenotypes are to be expected in healthy skin. The fluorescence properties of mixed phenotypes have not been studied.

Efforts to do so include their quantification in healthy human skin and in lesional and nonlesional skin of patients with skin diseases. Currently, the most common approach is to obtain skin biopsies and to visualize ΜΦs by immunohistochemistry. Skin biopsies, however, come with several important limitations, which include scarring, the risk of infection and bleeding, and artificial findings caused by the use of local anesthesia. In addition, histopathological analyses of skin biopsies are not well suited for characterizing ΜΦ functions such as phagocytosis and for long-term monitoring of ΜΦ distribution in the skin.

Fluorescence lifetime imaging (FLIM) employs NAD(P)H and fluorescence decay parameters of cellular compartments as specific indicators of cell types and phenotypes (Alfonso-García et al., 2016; Heaster et al., 2021). Combined with two-photon tomography, two-photon excited fluorescence lifetime imaging (TPE-FLIM) allows for label-free and noninvasive imaging of dermal cells. For instance, TPE-FLIM allows for in vitro imaging of mast cells, fibroblasts, neutrophils, and dendritic cells and in vivo imaging of mast cells in human skin (Kröger et al., 2020). Whether or not TPE-FLIM can be used to visualize human skin ΜΦs, their M1 and M2 phenotypes, and their functions, is currently unknown. There are, however, several independent lines of evidence that support this approach: First, previous studies have shown that TPE-FLIM can distinguish ΜΦs from other dermal cells and ECM, without prior labeling (Kröger et al., 2020). Second, the capillaries of the papillary dermis, which often are in close proximity to ΜΦs, show distinct TPE-FLIM signatures and are readily visualized (Shirshin et al., 2017). Third, M1 and M2 ΜΦs come with unique cytokine patterns, and the TPE-FLIM signatures of these cytokines and patterns could help to tell the two phenotypes apart. Finally, TPE-FLIM can distinguish between functional states of dermal cells, for example, resting and activated mast cells in vivo, ΜΦs ex vivo (Kröger et al., 2020), and T-cell activation in vitro (Walsh et al., 2021) may, therefore, potentially allow for monitoring ΜΦ functions in vivo (Szulczewski et al., 2016). Taken together, the morphological features of skin ΜΦs, their localization in the skin, and the expected differences in fluorescence decay parameters between ΜΦ phenotypes as well as between other dermal cells, make TPE-FLIM a promising strategy for their detection (Yakimov et al., 2019).

Here, we first investigated human skin ΜΦs, in vitro with clear M1 and M2 phenotypes, for their TPE-FLIM properties and how these differ from those of the main components of the ECM and other dermal cells such as fibroblasts, mast cells, and dendritic cells. We then applied the identified ΜΦ TPE-FLIM signatures to investigate M1 and M2 ΜΦs and their phenotypes in human skin biopsies, combined with traditional immunohistochemistry-based visualization. Finally, we used TPE-FLIM in vivo in humans to study skin ΜΦs, their phenotypes, and functions, and we developed, tested, and characterized TPE-FLIM signature-based machine learning algorithms for the detection of skin ΜΦs.

## Results

### In vitro monocyte-derived M1 and M2 ΜΦs show distinct TPE-FLIM parameters

**Figure 2.**
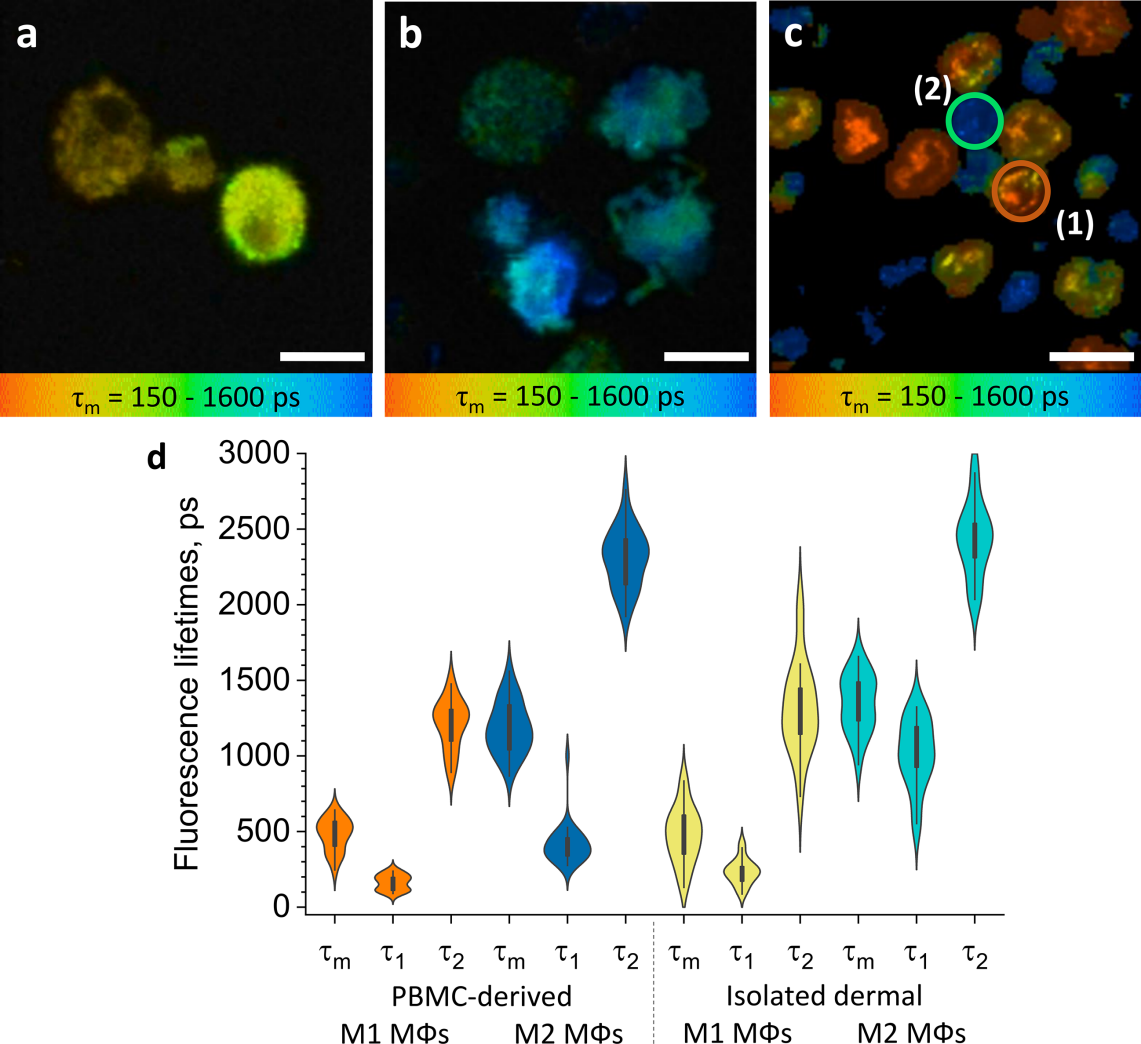
ΜΦs polarised from PBMC and isolated dermal ΜΦs show distinct TPE-FLIM signatures. TPE-FLIM *τ*_m_ images (mean fluorescence lifetime *τ*_m_ in the 150–1600 ps range) of monocyte-derived M1-polarised (IFN-γ) ΜΦs (**a**), monocyte-derived M2-polarised (IL-4) ΜΦs (**b**), and isolated human dermal M1 ΜΦs (1) and M2 ΜΦs (2) (**c**). Scale bar: 10 µm. The distribution of TPE-FLIM parameters *τ*_1_, *τ*_2_, and *τ*_m_ for monocyte-derived M1-polarised ΜΦs (*n*=21, orange), M2-polarised ΜΦs (*n*=27, dark blue), and isolated dermal M1 ΜΦs (*n*=34, yellow), M2 ΜΦs (*n*=28, light blue) (**d**). The boxplot represents 25–75% of the values. PBMC, peripheral blood mononuclear cell; TPE-FLIM, two-photon excited fluorescence lifetime imaging.

The TPE-FLIM images of monocytes isolated from human peripheral blood mononuclear cells (PBMCs) showed a round morphology (diameter of up to 10 µm) with a barely visible nucleus, homogeneously distributed cell content, and regular borders with no membrane extensions (Figure 2—figure supplement 1). ΜΦs differentiated from PBMC and polarised toward M1 ΜΦs with interferon-γ (IFN-γ; *n=*21) and toward M2 ΜΦs with interleukin-4 (IL-4; *n*=27) were similar in size, ranging 10–12 µm (Figure 2a–c). M1 and M2 ΜΦs showed comparable overall TPE-AF intensities, but they differed significantly in several other features. M1 ΜΦs also showed numerous bright spots (typical size is 2–3 µm), likely vacuoles and mitochondria, had less visible borders, and exhibited higher TPE-AF intensity than M2 ΜΦs. In contrast, M2 ΜΦs were characterized by distinct borders with filopodia (Figure 2b; Figure 2—figure supplement 2), which were rarely seen in M1 ΜΦs (Figure 2a).

**Figure 2—figure supplement 1.**
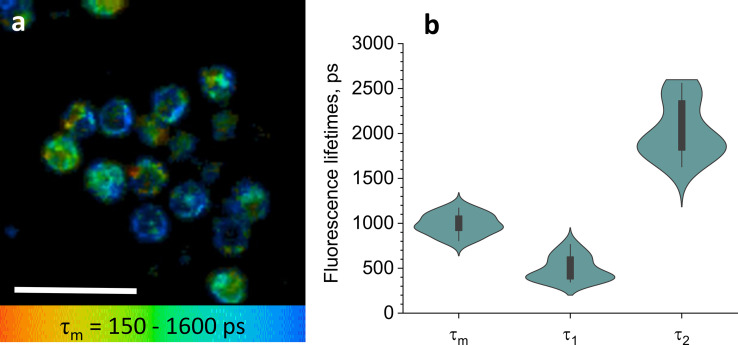
TPE-FLIM visualization of PBMC and histogram of TPE-FLIM parameter. TPE-FLIM *τ*_m_ images (mean fluorescence lifetime *τ*_m_ in the 150–1600 ps range) of PBMC (**a**) Scale bar: 10 µm. The distribution of TPE-FLIM parameters τ_1_, *τ*_2_, and τ_m_ for PBMC (*n*=15) (**b**).

**Figure 2—figure supplement 2.**
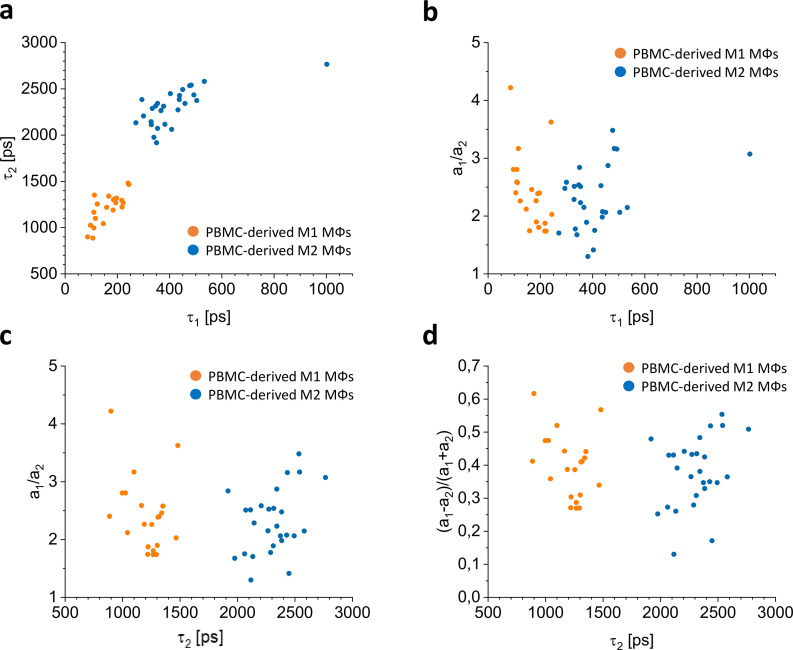
In vitro 2D segmentation for ΜΦs from PBMC. 2D segmentation of the *τ*_1_(*τ*_2_) (**a**), *τ*_1_(*a*_1_/*a*_2_) (**b**), *τ*_2_(*a*_1_/*a*_2_) (**c**), and *τ*_2_((a_1_–a_2_)/(*a*_1_+*a*_2_)) (**d**) TPE-FLIM parameters of the M1 ΜΦs (*n*=21, orange circles) and M2 ΜΦs (*n*=27, blue circles) measured in vitro.

**Figure 2—figure supplement 3.**
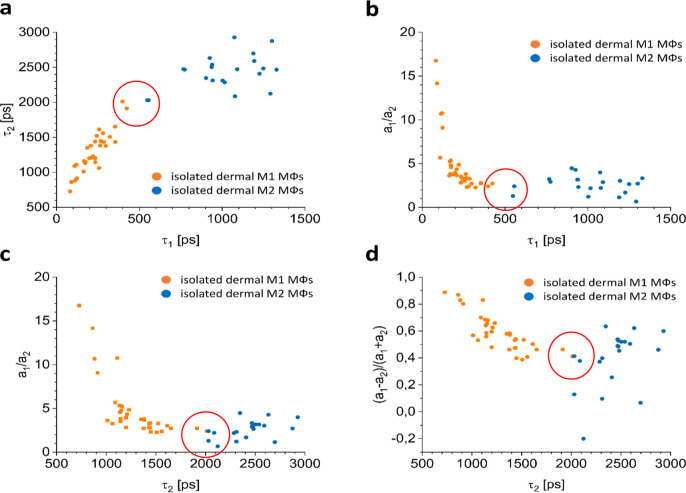
In vitro segmentation for ΜΦs from dermal tissue. 2D segmentation of the *τ*_1_(*τ*_2_) (**a**), *τ*_1_(*a*_1_/*a*_2_) (**b**), τ_2_(*a*_1_/*a*_2_) (**c**), and *τ*_2_((a_1_–a_2_)/(*a*_1_+*a*_2_)) (**d**) TPE-FLIM parameters of the M1 ΜΦs (*n*=34, orange circles) and M2 ΜΦs (*n*=28, blue circles) measured in vitro. Cells shown in red-marked areas were misidentified by the decision tree.

**Figure 2—figure supplement 4.**
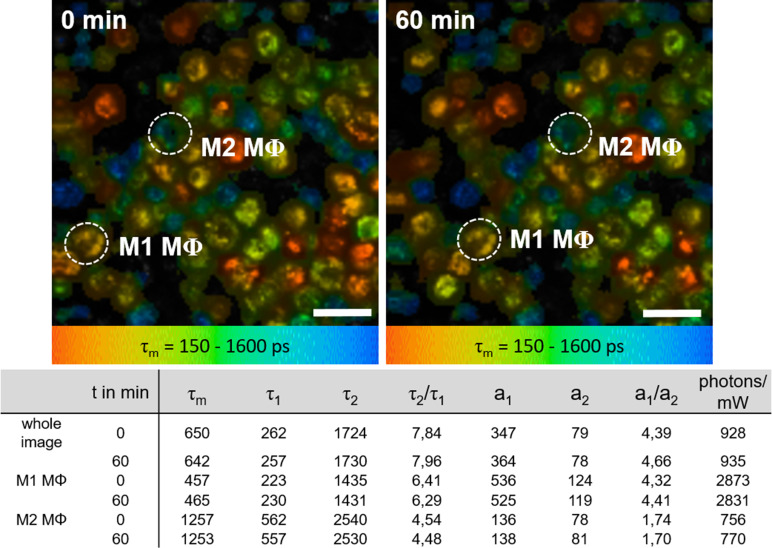
Time stability of TPE-FLIM parameters for ΜΦs isolated from periocular skin in vitro. TPE-FLIM images of M1 and M2 ΜΦs in vitro (shown in dotted ovals) showing mean fluorescence lifetime *τ*_m_ in color gradient from 150 to 1600 ps recorded with the time interval of 60 min. Scale bar: 15 µm. The images were recorded at 760 nm excitation wavelength, 6 mW laser power and 6.8 s acquisition time. Additionally, the TPE-FLIM parameters shown below the images are in agreement with M1 and M2 ΜΦs shown in the Table 1.

M1 and M2 ΜΦs also differed in their TPE-FLIM parameters *τ*_1_, *τ*_2_, and *τ*_m_ (Figure 2d). TPE-AF decay times were significantly shorter in M1 ΜΦs (*n*=21) than in M2 ΜΦs (*n*=27; *p*<0.05), and both ΜΦs differed significantly, in their TPE-FLIM parameters, from monocytes (*n*=15; *p*<0.05; Table 1).

**Table 1.** TPE-FLIM parameters for investigated dermal and epidermal cells. TPE-FLIM parameters *τ*_1_, *τ*_2_, *τ*_m_, *a*_1_/*a*_2_ and TPE-AF intensity of monocyte-derived M1 and M2 ΜΦs; dermal M1 and M2 ΜΦs isolated from the skin measured in vitro; M1 (CD68) and M2 (CD163) ΜΦs measured ex vivo in human skin cryo-sections; M1 and M2 ΜΦs observed on the forearm of healthy volunteers in vivo; monocytes; resting and activated human skin mast cells; dendritic cells; fibroblasts and neutrophils in vitro.

			Number of cells	*τ*_m_ in ps	*τ*_1_in ps	*τ*_2_in ps	*a*_1_/*a*_2_	TPE-AF intensity, photons /mW
**in vitro**	**Monocyte-derived M1-polarised ΜΦs**		21	479±106	163±50	1,209±161	2.4±0.6	600±100
**in vitro**	**Monocyte-derived M2-polarised ΜΦs**		27	1,185±170	417±134	2,305±194	2.3±0.5	500±100
**in vitro**	**M1 isolated dermal ΜΦs**		34	461±175	225±84	1,289±278	4.8±3.4	3,000±500
**in vitro**	**M2 isolated dermal ΜΦs**		28	1,281±155	807±250	2,352±229	2.2±1.1	800±200
**ex vivo**	**M1 ΜΦs** (**CD68**)		8	458±50	190±38	1,504±133	4.1±0.7	3,000±500
**ex vivo**	**M2 ΜΦs (CD163**)		12	1,369±201	498±129	2,267±155	1.1±0.4	700±300
**in vivo**	**M1 ΜΦs**		35	477±105	196±40	1,698±172	5.0±2.8	686±165
**in vivo**	**Phagocytosing** **M1 ΜΦs**		2	195±44	105±10	1,272±89	14.7±4.5	1,100±150
**in vivo**	**M2 ΜΦs**		25	1,407±60	442±54	2,458±90	1.2±0.2	360±155
**in vitro**	**PBMC-derived monocytes**		15	989±111	491±130	2,025±301	1.8±0.5	700±130
**in vitro**	**Resting mast cells**		43	1,248±287	533±266	2,289±317	1.5±0.5	1,300±400
**in vitro**	**Activated mast cells**		13	862±268	288±130	1,920±287	2.5±2.0	900±200
**in vitro**	**Dendritic cells**		14	1,265±180	434±188	2,578±328	1.6±0.2	538±258
**in vitro**	**Fibroblasts**		6	921±81	429±51	1,983±137	0.5±0.1	469±137
**in vitro**	**Neutrophils**		21	1,074±109	714±250	1,795±600	1.5±0.5	500±115
								

IgG stimulation of IgG immune complex-sensitized M1 and M2 ΜΦs resulted in the release of inflammatory mediators, but did not lead to significant changes or reveal additional differences in TPE-FLIM parameters 2 and 5 days after differentiation of PBMC into ΜΦs (data not shown). Taken together, these findings indicate that monocyte-derived M1 ΜΦs and M2 ΜΦs can be identified and distinguished in vitro by their distinct TPE-FLIM signatures.

### ΜΦs isolated from periocular skin show TPE-FLIM parameters that are similar to those of in vitro monocyte-derived M1 ΜΦs or M2 ΜΦs

Human ΜΦs isolated from periocular skin and analyzed by immunohistochemistry were irregularly shaped, with poorly defined borders, 8–10 µm in size, pericentral nuclei of 5–6 µm diameter with low fluorescence intensity, heterogeneously and irregularly distributed cellular content, and they exhibited a bright fluorescence multivacuolated cytoplasm with ≈1 µm diameter small bright spots, presumably related to mitochondria and/or vacuoles (Figure 2c). Based on their TPE-FLIM parameters, dermal ΜΦs fell into two significantly different groups (Figure 2d): group 1 (*n*=34), with stronger TPE-AF intensity (≈3000±500 photons/mW) and shorter lifetimes, and group 2 (*n*=28), with a weaker TPE-AF intensity (≈800±200 photons/mW) with longer lifetimes (Figure 2c; Figure 2—figure supplement 3). The profiles of dermal ΜΦs in groups 1 and 2 were similar to those of monocyte-derived M1 ΜΦs and M2 ΜΦs, respectively (Figure 2d; Table 1). The biggest differences between group 1/M1 ΜΦs and group 2/M2 ΜΦs were shorter *τ*_1_ and *τ*_m_ as well as larger size (10.9±0.6 µm) in the former as compared to the latter (9.8±1.2 µm; *p*<0.05; Figure 2c and d; Table 1). This suggests that human skin ΜΦs, based on their in vitro TPE-FLIM signatures, can be assigned to one of two phenotypes, where the first is similar to that of monocyte-derived M1 ΜΦs and the second is similar to that of monocyte-derived M2 ΜΦs.

It should be noted that the TPE-FLIM parameters were stable over the measurements that took up to 1 hr for in vitro M1 and M2 ΜΦs isolated from periocular skin (Figure 2—figure supplement 4). Their TPE-FLIM values vary within the standard deviation shown in Table 1.

### TPE-FLIM can distinguish between ΜΦs and other cells

To prove that the recorded TPE-FLIM signatures are unique for M1- and M2-polarized ΜΦs (Figure 2), we performed TPE-FLIM measurements of other dermal cells in vitro, such as mast cells, dendritic cells, fibroblasts, monocytes, and neutrophils. Their TPE-FLIM parameters, summarized in Table 1, are markedly different from those of the established signatures of M1- and M2-polarized ΜΦs. Thus, in addition to size, morphology, and internal vacuole structure, M1- and M2-polarized ΜΦs can be distinguished, from each other and other cells, by distinct TPE-FLIM parameters, a prerequisite for the visualization of skin ΜΦs ex vivo and in vivo. Table 1 is an extension of the results shown in Kröger et al., 2020.

### Immunohistochemistry confirms TPE-FLIM detection of M1 and M2 ΜΦs in human skin ex vivo

**Figure 3.**
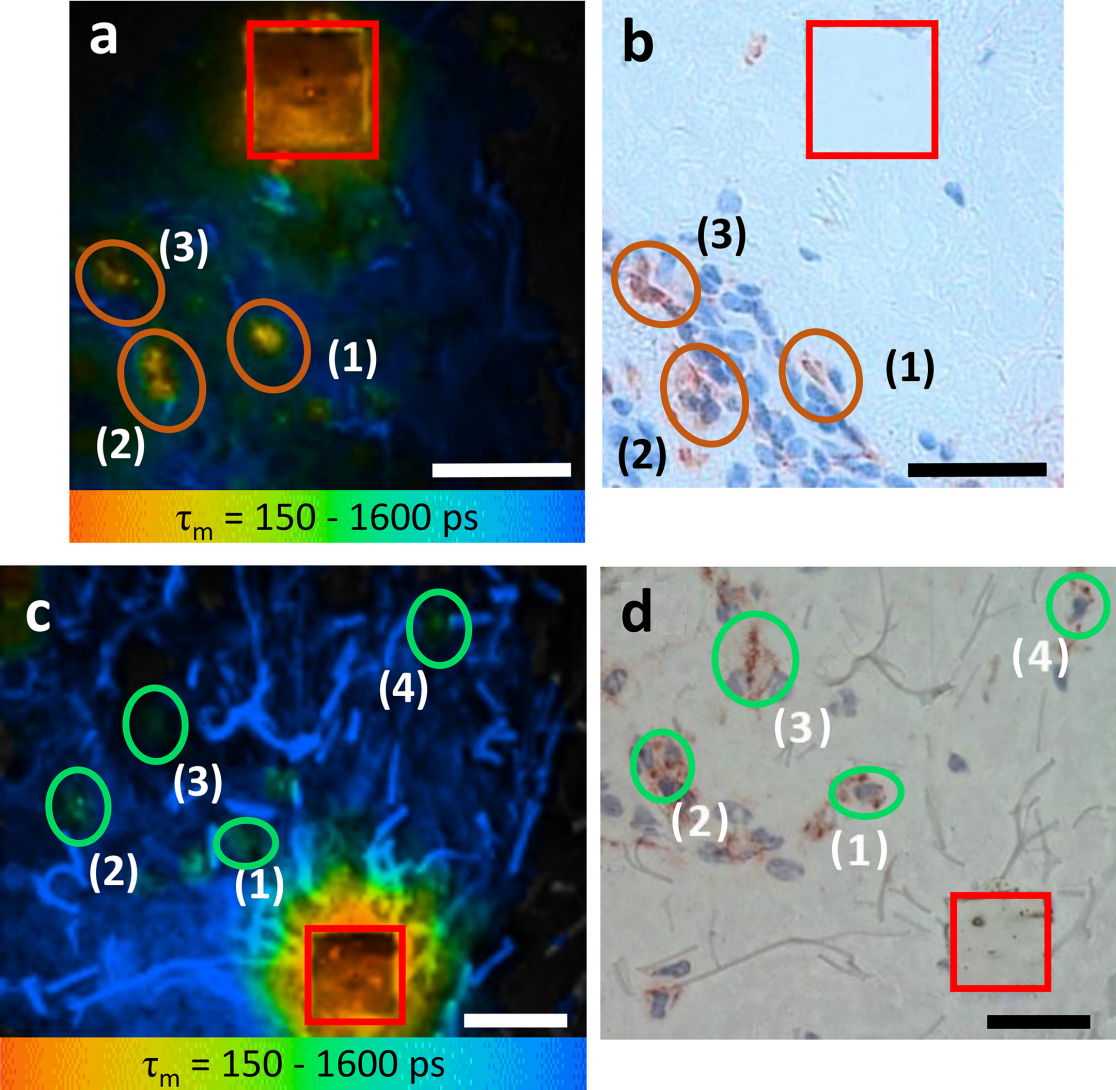
M1 and M2 ΜΦs ex vivo verified using TPE-FLIM parameters and immunohistochemistry-based bright field microscopy. Side by side comparison of TPE-FLIM τ_m_ images (mean fluorescence lifetime τ_m_ in the 150–1600 ps range), which were measured label-free and then stained with CD68-antibody for M1 ΜΦs (**a**), and CD163-antibody for M2 ΜΦs (**c**) and corresponding bright field microscopic images (**b**) and (**d**). The excitation wavelength is 760 nm and laser power is 4 mW (**a**) and 2 mW (**c**). The M1 and M2 ΜΦs are marked with ellipses in (**a, b**) and in (**c, d**), respectively. The laser-burned labels (28×28 µm^2^) are marked in red. The suspected (**a, c**) and staining-proved (**b, d**) ΜΦs are marked with number (1, 2, 3, and 4). More M2 ΜΦs are observed in (**d**) compared to (**c**) due to the staining and visualization of the entire biopsy volume in (**d**) and limited imaging plane of the two-photon tomograph (1.2–2.0 µm) in (**c**). Images have been rotated and zoomed to match their orientation and size. Scale bar: 30 µm. TPE-FLIM, two-photon excited fluorescence lifetime imaging.

To test if the TPE-FLIM signatures established in vitro identify M1- and M2-polarized ΜΦs in human skin, we sequentially analyzed dermal biopsies by TPE-FLIM and conventional immunohistochemistry. The application of in vitro ΜΦ signatures to TPE-FLIM analyses of 13 human skin biopsy cryo-sections identified two distinct cell populations: The first showed a short mean fluorescence lifetime *τ*_m_ and high TPE-AF intensity, a feature of M1 ΜΦs (Table 1, Figure 2d, Figure 3a); the second population showed longer *τ*_m_ and significantly lower TPE-AF intensity, typical for M2 ΜΦs (Table 1, Figure 2d, Figure 3c). CD68-staining for M1 ΜΦs and CD163-staining for M2 ΜΦs confirmed that short *τ*_m_ cells with high TPE-AF intensity were, indeed, M1 ΜΦs (Figure 3b) and that cells with longer *τ*_1_*, τ*_2_, and *τ*_m_ with low TPE-AF intensity were, indeed, M2 ΜΦs (Figure 3d, Figure 3—figure supplement 1, Table 1).

**Figure 3—figure supplement 1.**
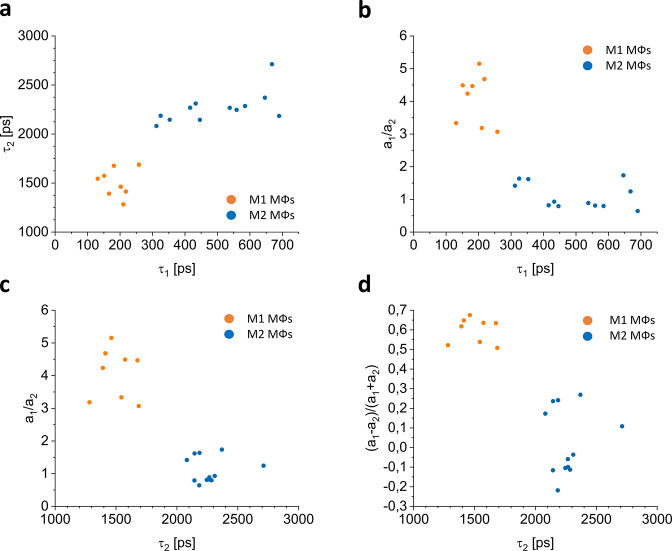
Ex vivo segmentation for ΜΦs from skin biopsies. 2D segmentation of the *τ*_1_(*τ*_2_) (**a**), *τ*_1_(*a*_1_/*a*_2_) (**b**), *τ*_2_(*a*_1_/*a*_2_) (**c**), and *τ*_2_((a_1_–a_2_)/(*a*_1_+*a*_2_)) (**d**) TPE-FLIM parameters of the M1 ΜΦs (*n*=8, orange circles) and M2 ΜΦs (*n*=12, blue circles) measured ex vivo.

**Figure 3—figure supplement 2.**
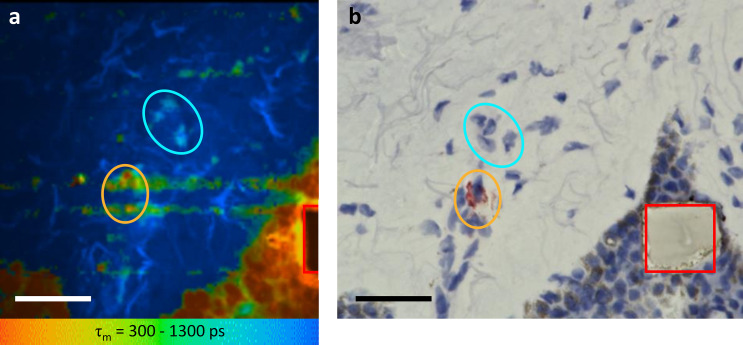
Mast cell-specific staining with tryptase ex vivo – negative control. Side by side comparison of TPE-FLIM image (average lifetime τ_m_ in the 300–1300 ps range) (**a**) and corresponding light microscopic image after staining of human skin biopsy cryo-sections for MCs (**b**). Laser power is 3 mW, excitation at 760 nm. The MCs are marked with orange ellipses, suspected ΜΦs are marked with blue ellipses, and the laser burned squares (28×28 µm^2^) used for labeling are marked in red. Images have been rotated and zoomed to match the same orientation and size. Scale bar: 30 µm.

CD68-positive dermal M1 ΜΦs showed a heterogeneous appearance, ranging from flat and spindle-shaped vessel lining to big intravascular with irregular borders and an irregular nucleus (Figure 3b). The TPE-FLIM image of CD163-positive M2 ΜΦs show round to elliptically shaped cells with a significantly lower TPE-AF intensity (Figure 3d). Of nine cells with a TPE-FLIM M1 ΜΦ signature, eight cells stained positive for CD68, and all CD68-positive cells had a TPE-FLIM M1 ΜΦ signature. As for M2 ΜΦs, all cells with a TPE-FLIM M2 ΜΦ signature (12 of 14) were CD163-positive, and all CD163-positive cells had a TPE-FLIM M2 ΜΦ signature.

### TPE-FLIM visualizes human skin M1 and M2 ΜΦs in vivo

**Figure 4.**
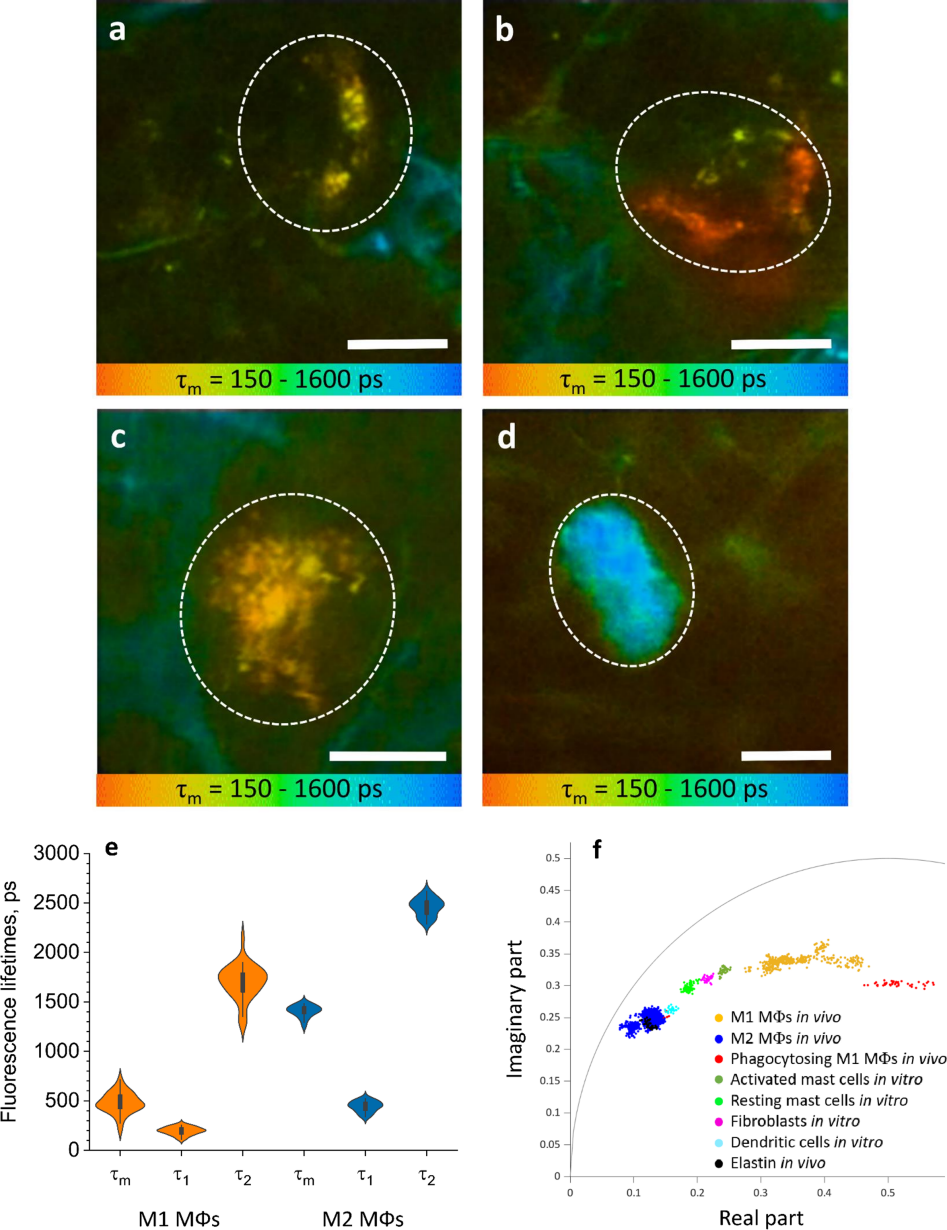
ΜΦs are visualized and categorised by TPE-FLIM signatures in vivo. TPE-FLIM in vivo images of potential perivascular flat spindle shaped M1 ΜΦ (**a**), of suspected slightly dendritic M1 ΜΦ in the depth 90 µm (**b**) large intervascular M1 ΜΦ with membrane extensions (**c**) and in vivo dermal cells resembling M2 ΜΦ were observed with a significantly longer mean fluorescence lifetime *τ*_m_ compared to M1 ΜΦs and less pronounced TPE-AF intensity (**d**), showing mean fluorescence lifetime *τ*_m_ in color gradient from 150 to 1600 ps. Scale bar: 10 µm. The histogram shows the distribution of TPE-FLIM parameters for M1 ΜΦs (*n*=35, orange) and M2 ΜΦs (*n*=25, blue) measured in vivo in human skin (**e**). The boxplot represents 25–75% of the values. The phasor plot has a threshold at 0.9 of the maximum intensity and shows a summary of 12 M1, 2 phagocytosing M1 ΜΦs and 12 M2 ΜΦs in vivo (**f**), where M1 ΜΦs are in orange and M2 ΜΦs in blue and phagocytosing M1 ΜΦs in red, the other dermal components are shown from in vitro measurements. The in vivo images (**a–d**) were recorded at 760 nm excitation wavelength, 50 mW laser power and 6.8 s acquisition time, in the depth of 80–100 µm on the volar forearm skin area of 25 healthy human subjects. TPE-AF, two-photon excited autofluorescence; TPE-FLIM, two-photon excited fluorescence lifetime imaging.

Next, we used TPE-FLIM to assess the skin of 25 healthy individuals in vivo, and we identified and further characterized 35 and 25 ΜΦs with an M1 and M2 TPE-FLIM signature, respectively. In vivo, similar to biopsy sections, M1 and M2 ΜΦs were located in the papillary and reticular dermis at >80 µm depth (Figure 4) and showed a density of >100 ΜΦs/mm² (Figure 1c and d). M1 ΜΦs fell into three distinct groups and were either flat and spindle-shaped (Figure 4a), slightly dendritic (Figure 4b), or large and intervascular (Figure 4c). M2 ΜΦs, in human skin in vivo, were round and moderately dendritic (Figure 4d), and they had a higher TPE-AF intensity in vivo compared to the ECM, as previously reported in vitro (Malissen et al., 2014; Njoroge et al., 2001).

**Figure 4—figure supplement 1.**
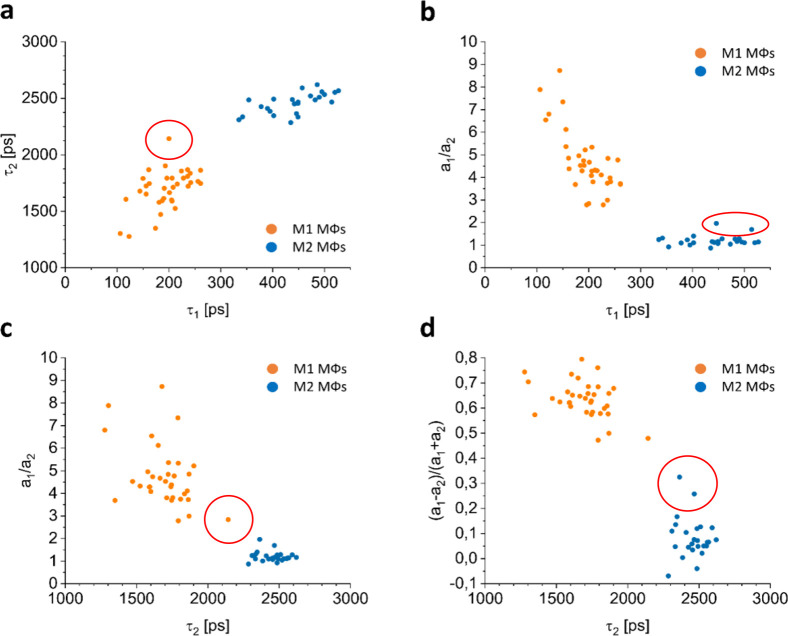
In vivo segmentation for ΜΦs in human skin. 2D segmentation of the *τ*_1_(*τ*_2_) (**a**), *τ*_1_(*a*_1_/*a*_2_) (**b**), *τ*_2_(*a*_1_/*a*_2_) (**c**), and *τ*_2_((a_1_–a_2_)/(*a*_1_+*a*_2_)) (**d**) TPE-FLIM parameters of the M1 ΜΦs (orange circles) and M2 ΜΦs (blue circles) measured in vivo. Cells shown in red-marked areas were misidentified by the decision tree.

**Figure 4—figure supplement 2.**
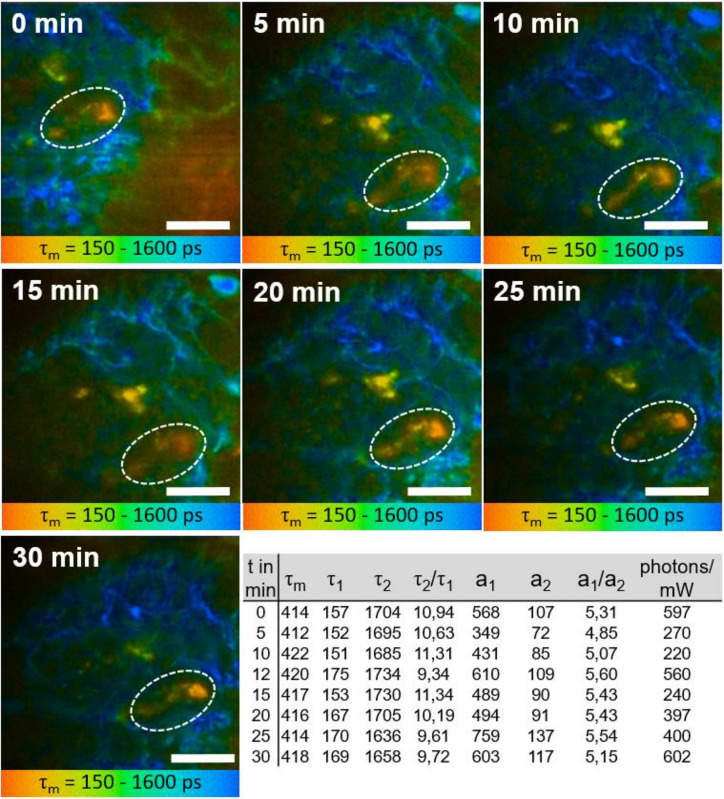
Time stability of TPE-FLIM parameter for M1 ΜΦ in vivo. TPE-FLIM images recorded in 5-min interval for 30 min of M1 ΜΦ measured in vivo in human papillary dermis at the depth 90 µm showing mean fluorescence lifetime *τ*_m_ in color gradient from 150 to 1600 ps. The M1 ΜΦ is marked with the white dotted ellipse. Scale bar: 20 µm. The images were recorded at 760 nm excitation wavelength, 50 mW laser power, and 6.8 s acquisition time on the plantar forearm. Additionally, the TPE-FLIM parameters shown below the images are in agreement with M1 ΜΦs shown in the Table 1.

**Figure 4—figure supplement 3.**
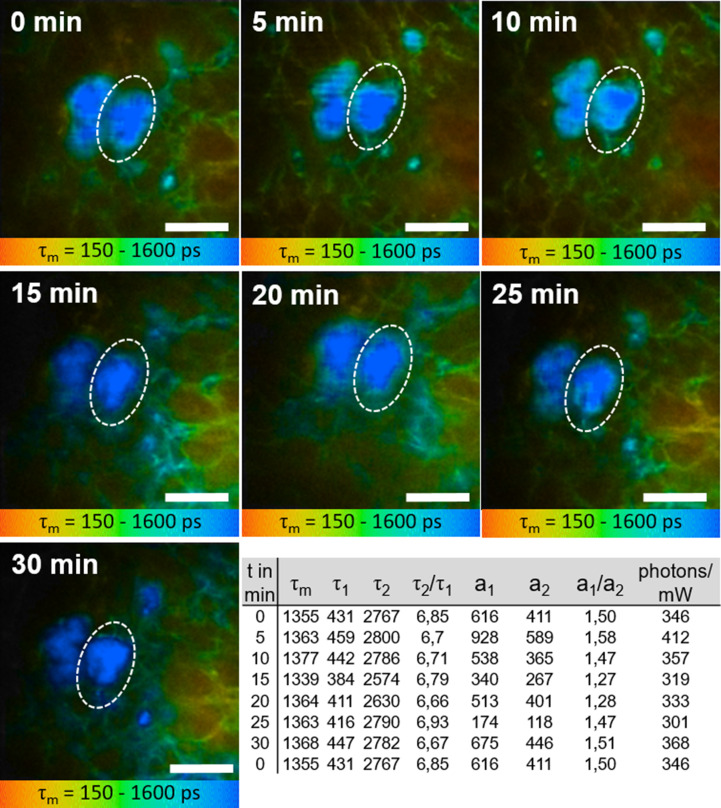
Time stability of TPE-FLIM parameter for M2 ΜΦ in vivo. TPE-FLIM images recorded in 5-min interval for 30 min of M2 ΜΦ measured in vivo in human papillary dermis at the depth 96 µm showing mean fluorescence lifetime *τ*_m_ in colour gradient from 150 to 1600 ps. The M2 ΜΦ is marked with the white dotted ellipse. Scale bar: 20 µm. The images were recorded at 760 nm excitation wavelength, 50 mW laser power, and 6.8 s acquisition time on the plantar forearm. Additionally, the TPE-FLIM parameters shown below the images are in agreement with M2 MΦs shown in the Table 1.

**Figure 4—figure supplement 4.**
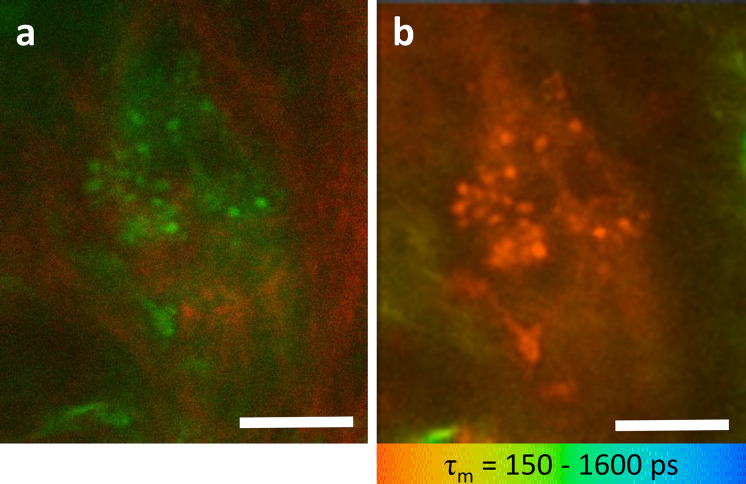
TPE-FLIM allows for visualization of potentially phagocytosing M1 ΜΦs in vivo. Merged TPE-AF (green) and SHG (red) image of possibly activated (phagocytosing) M1 ΜΦ measured in vivo in human papillary dermis at the depth 105 µm (**a**) and corresponding TPE-FLIM image (**b**), showing mean fluorescence lifetime *τ*_m_ in color gradient from 150 to 1600 ps. Scale bar: 10 µm. The images were recorded at 760 nm excitation wavelength, 50 mW laser power, and 6.8 s acquisition time on the plantar forearm.

**Figure 4—figure supplement 5.**
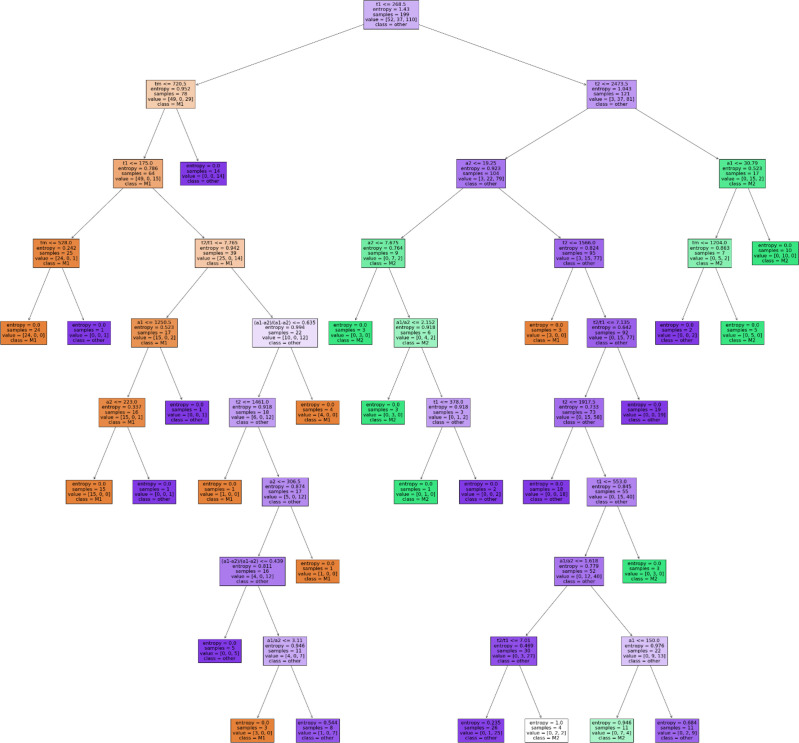
Decision tree model. Decision tree model for classification of M1 and M2 ΜΦs with the parameters entropy impurity, minimal samples for split is 2, maximum depth of the tree is 9, and the samples had equal weight. The text of the decision tree is readable when zoomed in.

**Figure 4—figure supplement 6.**
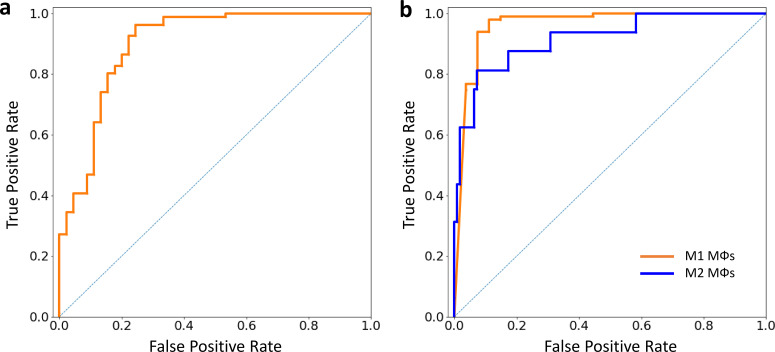
ROC curves of decision tree models. ROC curves of false positive to false negative rates for classification of ΜΦs versus other dermal cells (**a**) and for classification of M1 ΜΦs (orange line) versus M2 ΜΦs and other dermal cells and for classification of M2 ΜΦs (blue line) versus M1 ΜΦs and other dermal cells (**b**) generated from the decision tree classifier (Figure 4—figure supplement 5).

The TPE-FLIM parameters of in vivo M1 ΜΦs were in agreement with those of in vitro monocyte-derived and dermal M1 ΜΦs and ex vivo M1 ΜΦs (Figure 4e, Table 1). M2 ΜΦs in vivo have longer *τ*_m_ fluorescence lifetimes compared to in vitro and ex vivo experiments. Yet, the *τ*_1_ and *τ*_2_ were in agreement with in vitro PBMC-derived monocytes, and the size and morphological parameters were in line with what is expected in M2 ΜΦs. The 2D segmentation in Figure 4—figure supplement 1 shows the distinction of M1 and M2 ΜΦs presented in Figure 4a–d, and the phasor plot in Figure 4f shows that M1 and M2 ΜΦs could be distinguished from each other and from other dermal cells and ECM.

It should be noted that the TPE-FLIM parameters were stable over the measurements that took up to 30 min for in vivo M1 and M2 ΜΦs in the skin (Figure 4—figure supplements 2 and 3). The TPE-FLIM values vary within the standard deviation shown in Table 1.

### TPE-FLIM can potentially distinguish resting from phagocytosing human skin M1 ΜΦs in vivo

Phagocytosing skin M1 ΜΦs are characterized by an increase in cell size (May and Machesky, 2001), enhanced vacuolization (Cheng et al., 2019), a shift of TPE-FLIM parameters toward shorter fluorescence lifetime values (Yakimov et al., 2019), acidification (Li et al., 2017; Teixeira et al., 2018) and thus stimulated ROS production, different from those of resting M1 and M2 ΜΦs. A dermal cell matching all these criteria indicating phagocytosis is visualized in vivo using TPE-FLIM and presented in Figure 4—figure supplement 4. This cell is located in the reticular dermis, has an enlarged size (≈25 µm) and an oval shape, similar to the resting M1 ΜΦ in Figure 4c, pronounced vacuole structure and short TPE-FLIM lifetime indicative for phagocytosing M1 ΜΦ. Of 37 dermal M1 ΜΦs analyzed in vivo, 2 showed possible phagocytosis activity, and both were located in the reticular dermis below 100 µm of depth.

### Classification algorithm to identify ΜΦs in the skin

To separate M1 and M2 ΜΦs from other dermal cells, we developed a classification algorithm, which used the decision tree (Figure 4—figure supplement 5) and automatically classified ΜΦs based on their TPE-FLIM parameters and morphological features. The parameters of the decision tree were improved using hyperparameter optimization. The splitting method in the nodes of the decision tree classifier is chosen to be entropy impurity. To ensure the optimal quality of a split in the node of the decision tree, the following requirements had to be fulfilled: the minimal samples for a split are 2, the maximum depth of the tree is 9, and the samples had equal weight for the model classifying M1 and M2 ΜΦs. The independent TPE-FLIM parameters *τ*_1_, *τ*_2_, *a*_1_, and *a*_2_ and the dependent TPE-FLIM variables *τ*_m_, *τ*_2_/*τ*_1_, *a*_1_/*a*_2_, (*a*_1_*−a*_2_)/(*a*_1_+*a*_2_) have been used for the best classification results, as can be seen in the decision tree model in Figure 4—figure supplement 5. The ground truth was established by classification of in vitro and ex vivo ΜΦs with known phenotype resulting in 0.95±0.05 sensitivity and 0.97±0.06 specificity. When ΜΦs were classified as one group against other dermal cells, the sensitivity was 0.81±0.03 and the specificity was 0.81±0.03. Our algorithm also distinguished M1 ΜΦs from M2 ΜΦs and other cells, with a sensitivity of 0.88±0.04 and a specificity of 0.89±0.03. For distinction of M2 ΜΦs from M1 ΜΦs and other cells, the sensitivity was 0.82±0.03 and the specificity was 0.90±0.03; receiver operating characteristic (ROC) is shown in Figure 4—figure supplement 6. Additionally, a fivefold cross-validation was additionally executed with these results: (0.87; 0.92; 0.87; 0.89; and 0.94), the mean of k-fold scores using cross_val_score method is 0.90 with a score of 1 describing evenly distributed data.

## Discussion

This is the first in vivo study to show that human skin ΜΦs can be distinguished from other dermal cells and quantified through visualization with label-free, completely noninvasive TPE-FLIM. This risk-free approach also allows for the identification of ΜΦ phenotypes, that is, M1 and M2 ΜΦs, and for the characterization of their functional stage, that is, resting versus phagocytosing M1 ΜΦs. Finally, TPE-FLIM can be used to implement sensitive and specific machine learning algorithms for ΜΦ detection in the skin.

Our initial work with CD-14 positive monocytes isolated from PBMC and then differentiated and polarised toward M1 (IFN-γ) and M2 (IL-4) ΜΦs was needed to establish their TPE-FLIM parameters. In fact, it showed that ΜΦs are fluorescence-active, and, more importantly, that their TPE-FLIM parameters are among the best differentiators of M1 (*τ*_m_=479±106) and M2 (*τ*_m_=1,185±170) ΜΦs. M1 ΜΦs are associated with a slightly higher TPE-AF intensity (Table 1), which is a prominent indicator for the metabolic stress of the cell on account of a shift in lifetimes by changing amounts of free and bound NAD(P)H (AlShabany et al., 2016) and generation of ROS in mitochondria, phagosomal vacuoles, and the cell membrane (Datta et al., 2015). Additionally to NAD(P)H, autofluorescence of lipids and other cell compartments was recorded. TPE-AF intensity is a parameter with limitation due to the nonlinear imaging technique. There is no linear correlation between excitation and emission intensity, also it is reduced due to scattering and absorption in the skin. The metabolism of LPS-induced M1 ΜΦs is characterized by higher glycolysis, indicating a shift toward shorter fluorescence lifetime (Li et al., 2020; Orihuela et al., 2016). The longer fluorescence lifetime *τ*_2_ in M2 ΜΦs is best explained by oxidative phosphorylation and the emergence of fluorophores caused by fatty acid oxidation (Viola et al., 2019). NAD(P)H fluorescence is ubiquitously present in cells and exhibits the continuum of lifetimes in the 360–3400 ps range. Therefore, changes in TPE-FLIM parameters are likely a reason of the metabolic changes of the ΜΦs. Free NAD(P)H has a short lifetime of 360 ps. For bound NAD(P)H, longer lifetimes up to 2–4 ns have been reported (Alfonso-García et al., 2016). A higher ratio of bound to free NAD(P)H is associated with M2 MΦs resulting in longer TPE-FLIM parameters, while a lower ratio of bound to free NAD(P)H is associated with M1 MΦs resulting in faster TPE-AF decay (Blacker et al., 2014). Thus, a strong indicator for the ΜΦ polarization is the TPE-FLIM parameters of monocytes in between cohorts of ΜΦs.

It was observed that the quantity of fluorescence lifetimes in ΜΦs is vastly varying between M1 and M2 ΜΦs. Regarding the ΜΦ polarization, the paradigm shifts toward a less strict classification compared to M1 (IFN-γ/LPS-polarized) and M2 (IL-4-polarized). While this categorization is useful in clinical terms, the multitude of parameters leading to the differentiation process leaves ΜΦs with wide-ranging properties both in expression of markers and also in appearance and TPE-FLIM parameters (Murray, 2017).

M1 (IFN-γ/LPS-polarized) ΜΦs rely on the NADH oxidase and production of ROS, which is shown by fluorescent lifetimes of under 250 ps and mitochondrial fission, which can indicate the bright spots, whereas M2 (IL-4-polarized) rely on oxidative phosphorylation and fatty acid oxidation, together with mitochondrial fusion, it can explain the homogeneous appearance of M2 ΜΦs (Ramond et al., 2019; Swindle et al., 2002; Xu et al., 2016).

Translation of our in vitro findings to ΜΦs isolated from human skin confirmed that the latter share the TPE-FLIM signatures of the former, with shorter *τ*_m_ in dermal M1 ΜΦs and longer *τ*_m_ in dermal M2 ΜΦs. The classification into M1 and M2 ΜΦs in vitro based on their distinct TPE-FLIM parameters was supported by their differences in size, morphology and internal vacuole structure. That the *τ*_1_ lifetime of dermal M2 ΜΦs is longer than that of monocyte-derived M2 ΜΦs is most likely due to the use of different polarization agents. ΜΦ colony-stimulating factor (M-CSF) and IFNγ for M1 ΜΦs and ΜΦ colony-stimulating factor (M-CSF) and IL-4 for M2 ΜΦs were used in PBMC-derived ΜΦs and microenvironment effects, like inflammatory signals, UV exposure (Kang et al., 1994), and immune responses (Theret et al., 2019) influencing ΜΦ functions, result in divergent fluorescence lifetimes (Zhang et al., 2014; Table 1, Figure 2d). The most important outcome of our work with dermal MΦs was the establishment of their phenotype-specific TPE-FLIM signatures, a prerequisite for our subsequent in vivo studies and for comparing skin MΦs and other dermal cells.

In fact, the use of the TPE-FLIM signatures of M1 and M2 ΜΦs clearly allowed to distinguish them from mast cells, dendritic cells, fibroblasts, and neutrophils (Table 1, Figure 4f; Kröger et al., 2020). We controlled this by independent markers. For example, ΜΦs exhibited a higher fluorescence intensity compared to other dermal cells. In addition, they were larger than dermal mast cells, and their morphology was clearly different from that of neutrophils and fibroblasts. Vacuoles, a defining feature of ΜΦs, were linked to ΜΦ TPE-FLIM parameters, whereas granules, which identify mast cells, were not. In short, dermal ΜΦs, in their TPE-FLIM profiles, do not superimpose with other dermal cells or structures. The only exception, a partial overlap between M2 ΜΦs and elastin, is not relevant for the visualization of the former, as they are readily distinguished from the latter based on their morphology.

To confirm that ex vivo TPE-FLIM overlap with TPE-FLIM signatures of M1 and M2 ΜΦs in vitro, we sequentially analyzed cells in skin biopsy cryo sections with TPE-FLIM and conventional immunohistochemistry. Indeed, both approaches identified and distinguished matching ΜΦ populations, that is, M1 and M2 ΜΦs, with strong fluorescence intensity and spindle shape appearance of M1 ΜΦs and lower fluorescence intensity and longer fluorescence decay in M2 ΜΦs (Figure 3). Interestingly, M2 ΜΦs are often found in an area of higher density of unknown dermal cells, presumably fibroblasts, compared to M1 ΜΦs. It is suspected that M2 ΜΦs in conjunction with collagen-synthesizing fibroblasts are acting toward and aiding in dermal repair and regeneration. However, this approach also revealed some challenges that come with ΜΦ visualization by TPE-FLIM. For example, it was more difficult to visualize M2 ΜΦs than M1 ΜΦs in biopsies due to the high fluorescence intensity of elastin in dried tissue and other ECM components and a decreased signal-to-noise ratio. In Figure 3d, more CD163 positive M2 ΜΦs are visible compared to the corresponding TPE-FLIM image in Figure 3c, which is due to previously mentioned challenges and the limited imaging plane of the two-photon tomograph (1.2–2.0 µm) compared to a significantly thicker biopsy section (10 µm), which was stained in an entire depth and visualized by bright field microscopy. Importantly, immunohistochemistry confirmed our ΜΦ phenotype-specific TPE-FLIM signatures, and, in addition, confirmed that they distinguish ΜΦs from other dermal cells. Skin mast cells, for example, stained for tryptase, showed a distinct TPE-FLIM signature, confirming our recently reported findings on dermal mast cells in vivo (Kröger et al., 2020), and distinguished them from M1 and M2 ΜΦs (Figure 3—figure supplement 2).

When we turned to the visualization of skin ΜΦs in vivo, we had first to develop a search strategy. Important considerations included the preferred localization of ΜΦs in the papillary and reticular dermis, the orientation of the focal plane parallel to the skin surface, and the need for maximal cellular cross-section visualization, which requires a high-resolution adjustment in depth to reconstruct an entire cell structure. The application of this search algorithm successfully visualized M1 and M2 ΜΦs in human skin in vivo. The in vivo TPE-FLIM parameters of M1 ΜΦs were in agreement with those observed in vitro and ex vivo. M2 ΜΦs in vivo were characterized by longer mean fluorescence lifetime *τ*_m_ compared to in vitro and ex vivo (Table 1), which can be explained by the influence of environment (Koo and Garg, 2019; Njoroge et al., 2001). ΜΦs measured in vivo differ from cells measured in vitro by their simplified microenvironment (Mosser and Edwards, 2008) with missing growth factors and cytokines (Melton et al., 2015) and an elevated level of nutrients, which leads to different polarization of ΜΦs and different contributions of fluorescence lifetimes. Membrane extensions were harder to detect in vivo due to the obscuring effect of the surrounding ECM. We also observed that TPE-FLIM parameters in the same ΜΦs can vary depending on their cellular substructures, for example, the nucleus, vacuoles, cytoplasm, or membrane (Figure 4a–d). The phasor plot shows the relative position of the categories of ΜΦs and other cells. Furthermore, it shows the contributions of long and short fluorescence lifetimes, their discrepancies (Table 1) being due to the computational method and the harmonics at the repetition frequency of 80 MHz. Further investigations are needed to clarify how the location, morphology, and function of M1 and M2 ΜΦs influence their TPE-FLIM parameters and TPE-AF intensity in vivo. Such studies should also address the reasons for the differences in TPE-FLIM parameters between M1 and M2 ΜΦs, which may include differences in their metabolic pathways. M1 ΜΦs, for example, rely on NADH oxidase and production of ROS, which is linked to short fluorescence lifetimes below 250 ps and mitochondrial fission. M2 ΜΦs, on the other hand, rely on oxidative phosphorylation and fatty acid oxidation, together with mitochondrial fusion (Ramond et al., 2019; Swindle et al., 2002; Xu et al., 2016).

The ability to visualize M1 and M2 ΜΦs by TPE-FLIM in vivo also makes it possible to explore how and why ΜΦs’ morphology, location, and functions are linked. When activated, the cytoskeletal structure and cellular appearance of ΜΦs change, and this may also affect their TPE-FLIM parameters. M1 ΜΦs are elongated, with a dense actin network along the cortex. M2 ΜΦs are more spherical with more randomly distributed actin (Porcheray et al., 2005; Vogel et al., 2014; Zhang et al., 2014; Figure 4b and d). Actin reorganization in M1 and M2 ΜΦ polarization and activation lead to bigger filament bundles of the actin cytoskeleton, which reduces cell plasticity (Colin-York et al., 2019; Pergola et al., 2017). As reported by Vogel et al., 2014, ΜΦ migration in the skin depends on their polarization. M1 ΜΦs, due to changes in actin cytoskeleton, migrate less far than M2 ΜΦs. Our TPE-FLIM findings confirm this, as we detected M1 ΜΦs via their high fluorescence and short autofluorescence lifetimes primarily in close proximity to blood capillaries. The irregular appearance of M1 ΜΦ detected by TPE-FLIM is likely a consequence of polarization-specific changes of the cellular cytoskeleton (McWhorter et al., 2013). The only morphological feature observed in both, M1 and M2 populations of ΜΦs, is that they are moderately dendritic, possibly because such ΜΦs are in the process of polarization, prior to cytoskeletal changes (Sica and Mantovani, 2012), or because polarization in ΜΦs is reversible polarizing (Sica and Mantovani, 2012; Yuan et al., 2017). Future studies should characterize the influence of cytoskeletal changes on TPE-FLIM parameters in detail and use TPE-FLIM to assess the impact of age, gender, and disease on the ratio, localization, and function of M1 and M2 ΜΦs in the skin (Fukui et al., 2018).

Time stability measurements were performed in vitro on M1 and M2 ΜΦs isolated from periocular skin within 1 hr (Figure 2—figure supplement 4) and in vivo on M1 ΜΦ within 30 min (Figure 4—figure supplements 2 and 3).

TPE-FLIM parameters were stable and varied within the standard deviation range shown in Table 1. It was possible to visualize single cells over this long-term time period in vitro (Figure 2—figure supplement 4) and in vivo (Figure 4—figure supplements 2 and 3), yet no unprompted change in fluorescence lifetime was observed, given the laser power was not high enough to induce photoproducts or photobleaching of the fluorophores. The NAD(P)H-related TPE-FLIM parameter showed no change over the course of the measurement nor did the fluorescence intensity. The limitation of long-term in vitro measurements is that the cells cannot be heated and will eventually cool down to room temperature. The limitation of long-term in vivo measurements is inherent to the individual. It is technically possible to investigate one subject over the course of multiple hours, but it is almost impossible to find the same cell again in another measurement.

Another limitation of this study is the simplified separation between M1 and M2 ΜΦs without taking into consideration the mixed ΜΦ phenotype. As mentioned in the introduction, mixed phenotype of ΜΦs is primarily observed in diseases like melanoma, systemic sclerosis, Lupus, and in recovery from LPS tolerance. Thus, we do not expect a significant amount of mixed phenotype ΜΦs in healthy and asymptomatic skin. However, the ΜΦs misidentified by the decision tree (Figure 2—figure supplement 3 and Figure 4—figure supplement 1) could potentially be ΜΦs of a mixed phenotype, which should be proved in additional experiment.

During phagocytosis, the generation of ROS by NAD(P)H oxidase leads to the highest degree of metabolic stress observed in M1 ΜΦs besides apoptosis (Dupré-Crochet et al., 2013; Shirshin et al., 2019), and ROS localization in vacuoles in phagocytosing M1 ΜΦs as a bactericidal mechanism (Dupré-Crochet et al., 2013; Myers et al., 2003). This is why phagocytosing M1 ΜΦs change their TPE-FLIM lifetimes toward shorter values and their vacuoles become visible as localized bright spots, which makes their in vivo detection possible (Figure 4—figure supplement 4, Table 1; Cannon and Swanson, 1992). The results shown here for possible phagocytosis are supported by the appearance (large size) and shortened fluorescence lifetime values for erythrophagocyting cells, presented by our group (Yakimov et al., 2019), resembling the cell shown in this study. The microenvironment in inflamed skin is known to be acidic with a pH<7.35 (Haka et al., 2009). Acidification is also known in phagocytosing ΜΦs (Teixeira et al., 2018). Together with the fact that fluorescence lifetime of fluorophores is shifted toward shorter values (Li et al., 2017) and cells especially ΜΦs produce ROS under phagocytosis and acidic conditions (Slauch, 2011), we have very compelling indications for the visualization of phagocytosing M1 ΜΦs. The influence of different phagocytosed materials in ΜΦs should be investigated in the future. TPE-FLIM potentially allows for the detection of possible phagocytosing M1 ΜΦs and is used as a confounder in the classification but cannot detect what is phagocytized. No internal structure was visible in TPE-FLIM images. The precision of measurements of cells and structures with short fluorescence lifetimes, such as phagocytosing ΜΦs, could be improved by reducing the value of the instrument response function (IRF), which is <100 ps in our measurements.

The construction of the feature vector and the resulting hyperparameter optimized decision tree model (Figure 4—figure supplement 5) yielded proficient results for the automatised classification of M1 and M2 ΜΦs, demonstrating that M1 and M2 ΜΦs can be separated from each other and other cells in the skin with high accuracy, that is, sensitivity and specificity, without need of additional staining using a supervised machine learning approach. The decision tree model uses only the independent TPE-FLIM parameters *τ*_1_, *τ*_2_, *a*_1_, and *a*_2_ and the dependent TPE-FLIM variables *τ*_m_, *τ*_2_/*τ*_1_, *a*_1_/*a*_2_, (*a*_1_*−a*_2_)/(*a*_1_+*a*_2_). This indicates that macrophages could be distinguishable completely from other cells without the use of morphologic parameters, thus reducing the degree of freedom and saving calculation and annotation time by software and physicians. The high accuracy for M1 macrophages is owed to the fact that M1 ΜΦs have the shortest τ_1_ and τ_2_ fluorescence lifetimes, the highest ratio of a_1_/a_2_ and the highest fluorescence intensity of the cells in the model. M2 ΜΦs can be misclassified in rare cases with resting mast cells and in vitro dendritic cells. Concluding from these results that the classification is only dependent on TPE-FLIM parameters and not on the morphology of dermal cells.

Ideally the data sets consist of the same amount of entries for every three classes (M1 ΜΦs, M2 ΜΦs, and other cells). In the experimental reality, those three classes are not evenly distributed and could lead to overemphasize of certain classes in the classifier model. It is shown here by the methods above that the data set is useable for the decision tree classifier. Overfitting of the relatively small data set is avoided with the parameters for the decision tree model, first by randomly splitting the data set into training and test set 10,000 times resulting in a small standard deviation and with the use of k-fold cross-validation resulting in a mean of the cross-validation score of 0.90, which equates to 90% accuracy. A cross-validation score of 1 describes perfectly even distributed data in all folds. The robustness and accuracy of this approach can be improved further, by the introduction of a depth-adjusted cell size and refined cell shape parameters and by increasing the number of in vivo ΜΦs integrated into the algorithm and training data set.

## Materials and methods

### Two-photon excited fluorescence lifetime imaging

For imaging of human ΜΦs, a two-photon tomograph (Dermainspect, JenLab GmbH, Jena, Germany), equipped with a tunable femtosecond Ti:sapphire laser (Mai Tai XF, Spectra Physics, USA, 710–920 nm, 100 fs pulses at a repetition rate of 80 MHz), was used at 3–5 mW for measurements of cells in vitro and skin biopsy sections ex vivo, as well as human dermis in vivo at 40–50 mW. The excitation wavelength was set to 760 nm, and a 410–680 nm band pass filter was used to detect two-photon excited autofluorescence (TPE-AF), whereas a 375–385 nm band pass filter was used to detect the second harmonic generation signals. The axial and lateral resolution was approximately 1.2–2.0 and 0.5 µm, respectively (Breunig et al., 2013). The screening depth covers the entire papillary dermis and part of reticular dermis (Darvin et al., 2014; König, 2008; Kröger et al., 2020).

Fluorescence decay of a specimen was recorded and analyzed in the SPCImage 8.0 software (Becker&Hickl, Berlin, Germany). TPE-FLIM data were fitted with a bi-exponential decay function. The TPE-AF intensity threshold was chosen depending on the signal-to-noise ratio, minimizing noise in the region of interest. The shift of the signal in relation to the instrument response function (IRF) was compensated. The typical IRF value was <100 ps. The TPE-AF decay curves were averaged over the central pixel of the region of interest and the 48 closest square neighbouring pixels (binning=3), resulting in a number of detected photons for each fluorescence decay curve larger than 5000. The TPE-AF decay parameters, decay lifetimes (*τ*_1_ and *τ*_2_) and amplitudes (*a*_1_ and *a*_2_), were used for the evaluation of the fluorescence lifetime distributions and 2D segmentation (Shirshin et al., 2017). The analyzed parameters were the mean lifetime, defined as *τ*_m_=(*a*_1_*τ*_1_+*a*_2_*τ*_2_)/(*a*_1_*+a*_2_) and the ratios *τ*_2_/*τ*_1_, *a*_1_/*a*_2_ and (*a*_1_*−a*_2_)/(*a*_1_*+a*_2_), which were used for 2D segmentation analysis. The TPE-FLIM data were also analyzed and represented as phasor plots, that are based on the transformation of the fluorescence decay data in the frequency domain, whereas the decay is described as amplitude and phase values of the first Fourier component (Digman et al., 2008). The phasor plots’ *x*-axis is described by the cosine of the phase value multiplied by the amplitude, the *y*-axis represents the sine of the phase value multiplied by the amplitude (Lakner et al., 2017; Shirshin et al., 2019). The position of the mean lifetime is on the secant from *τ*_1_ and *τ*_2_, the distance to the circle is given by the proportion of *a*_1_ and *a*_2_. The TPE-FLIM data were normalized to the maximum intensity and the threshold of 70% was set when analysing the phasor plots. The comparison of the bi-exponential fitting and phasor analysis in separation between cells subpopulations when treating the FLIM data was analyzed in Shirshin et al., 2022—it this work, we used both approaches to separate the M1 and M2 macrophages.

### FLIM data processing

The fluorescence decay curves were fitted with the bi-exponential decay model. Justification of the choice of the model and its comparison to the three exponential fitting is presented in the SI (Section FLIM data analysis). The absence of correlations between the fluorescence intensity and fluorescence decay parameters, as well as for fitting quality (assessed as χ^2^) and fluorescence decay parameters was additionally verified as described in the SI.

### Ethical considerations and study conduct

Volunteers for intravital imaging provided their written informed consent before participation. Skin samples taken from periocular skin surgery for ΜΦ preparation and all human skin investigated in this study were used after written informed consent was obtained. Positive votes for the experiments have been obtained from the ethics committee of the Charité – Universitätsmedizin Berlin (EA1/078/18, EA4/193/18, and EA1/141/12), which were conducted according to the Declaration of Helsinki (59th WMA General Assembly, Seoul, October 2008).

### Study subjects

Twenty-five healthy volunteers (12 males and 13 females, 24–65 years old, skin type I–III according to Fitzpatrick, 1988 classification) with asymptomatic volar forearm skin without preexisting health conditions were randomly selected for noninvasive in vivo measurements in the papillary dermis using TPE-FLIM. Visually impairing hair was removed with a scissor prior to measurements. The oil immersion objective of the microscope was connected to the skin via a 150 µm thick, 18 mm diameter cover glass (VWR, Darmstadt, Germany) with a ≈10 µl distilled water droplet between cover glass and skin. About 6–12 in vivo tomograms (different skin areas) were measured per subject, the investigated volume is from ≈70 µm depth in the papillary dermis to ≈130 µm depth in the reticular dermis with an image size of 150×150 µm^2^. This adds up to (60×150×150) µm³ times 6–12 images, with a total volume of 0.008–0.016 mm³ of papillary and reticular dermis seen per subject, the average time spent was ≈30 min per subject and the acquisition time was 6.8 s per image. The volunteers were screened between October 2018 and November 2020.

### Investigation of human dermal ΜΦs in vitro

Human dermal ΜΦs were prepared from periocular tissue (Botting et al., 2017). Human periocular skin was digested in 2.4 U/ml dispase type II (Roche, Basel, Switzerland) at 4°C for 12 hr. The dermis was minced with scissors after removal of the epidermis and further digested in PBS containing Ca^2+^ and Mg^2+^ (Gibco, Carlsbad, CA) supplemented with 1% Pen/Strep, 5% FCS, 5 mM MgSO_4_, 10 µg/ml DNaseI (Roche), 2.5 µg/ml amphotericin (Biochrom, Berlin, Germany,) 1.5 mg/ml collagenase type II (Worthington Biochemical Corp, Lakewood, NJ), and 0.75 mg/ml H-3506 hyaluronidase (Sigma-Aldrich, St. Louis, MO) at 37°C in a water bath with agitation for 60 min. The cell suspension was filtered using 300 and 40 µm stainless steel sieves (Retsch, Haan, Germany). Centrifugation at 300×*g* for 15 min at 4°C was applied next. The digestion cycle was repeated once. ΜΦs were isolated by Pan Monocyte Isolation Kit (Miltenyi, Bergisch Gladbach, Germany) after washing in phosphate-buffered saline (PBS) w/o Ca^2+^ and Mg^2+^ (Gibco), and kept in basal Iscove’s medium supplemented with 1% Pen/Strep, 10% FCS, 1% non-essential amino acids, 226 µM *α*-monothioglycerol (all Gibco). For long-term cultures, after 24 hr recombinant human IL-4 (20 ng/ml) and hSCF (100 ng/ml) (both Peprotech, Rocky Hill, NJ) were added. Purity of ΜΦ cultures was routinely checked to be >85% (Nielsen et al., 2020). For imaging, cells were used after 3 days in medium, washed two times with PBS before seeding on 18 mm diameter microscope cover glass (VWR) for imaging in PBS containing Ca^2+^ (Gibco) at room temperature.

### Investigation of peripheral blood monocytes in vitro

Peripheral blood monocytes were isolated from human blood using 15 ml Ficoll-Paque (VWR) centrifugation gradient. Centrifugation was performed at 1000×*g* for 1 min, with added 9 ml heparin and filled to 50 ml with PBS. Centrifugation was then repeated at 1000×*g* for 10 min, discarding the upper plasma layer and collecting the PBMC layer. The cells were washed two times with PBS and centrifuged at 350×*g* for 10 min. The supernatant was discarded and cultured in 5 ml basal Iscove’s medium supplemented with 1% Pen/Strep, 10% FCS (Biochtrom, Berlin, Germany) and subsequently incubated at 37°C and 5% CO_2_ for 2 hr before seeded and imaged on an 18 mm diameter microscope cover glass (VWR) in PBS containing Ca^2+^ (Gibco) at room temperature.

### Investigation of ΜΦs differentiated from peripheral blood monocytes in vitro

ΜΦs were differentiated from peripheral blood monocytes and polarised into M1 (IFNγ)-like state with ΜΦ colony-stimulating factor (M-CSF) and IFNγ and M2 (IL-4)-like state with ΜΦ colony-stimulating factor (M-CSF) and IL-4. For further stimulation, cells were incubated with LPS at 37°C for 24 hr prior to imaging. Due to a simplified environment with specific differentiation agents, the differentiation of monocytes was partially incomplete. Exemplary monocyte-derived ΜΦs appearing as M1 or M2 ΜΦs were measured and analyzed by TPT/FLIM. The requirement for M1 ΜΦs was a granular appearance and for M2 ΜΦs a dendritic appearance.

### Investigation of dendritic cells in vitro

CD14 positive PBMCs were used to differentiate dendritic cells by washing in PBS and centrifuging at 350×*g* for 10 min two times. About 5 ml RPMI medium, supplemented with 1% Pen/Strep and 1% FCS (Biochtrom), was added. Tryptan Blue (Sigma-Aldrich) was used for counting the cells in a hemocytometer, seeded at 2.0×10^6^ cells/ml and incubated for 2 hr at 37°C under 5% CO_2_. Non-attached cells and the supernatant were discarded. Adding 500 µl basal Iscove’s medium to the cells supplemented with 1% Pen/Strep, 1% glutamine, 5% HSA (all Gibco), 100 ng/ml IL-4, 100 ng/ml GM-CSF (both Peprotech) with medium change every second day for 6 days at 37°C. For TPE-FLIM imaging, the cells were seeded on 18 mm diameter microscope cover glass (VWR) in PBS containing Ca^2+^ at room temperature.

### Preparation and cryo-sectioning of human skin for combined TPE-FLIM and histomorphometric analysis

Thirteen human skin biopsy cryo-sections were prepared and measured using the TPE-FLIM method to acquire TPE-FLIM parameters of suspected M1 and M2 ΜΦs. The skin biopsies were obtained from abdominal reduction surgery of four female patients (31, 33, 40, and 44 y. o., skin type II according to Fitzpatrick classification; Fitzpatrick, 1988). Punch biopsies of 6 mm diameter were obtained, frozen, and stored at –80°C before cryo-sectioning. Vertical histological cryo-sections of 10 µm thickness were prepared on a cryostat (Microm Cryo-Star HM 560, MICROM International GmbH, Walldorf, Germany) after embedding in a cryo-medium (Tissue Freezing Medium, Leica Biosystems Richmond Inc, Richmond, IL) and placed on 18 mm diameter microscope cover glasses (VWR). The anatomical condition of the biopsies was continuously examined using a transmission microscope (Olympus IX 50, Olympus K.K., Shinjuku, Tokyo, Japan).

Using TPE-FLIM, cryo-sections were searched for cells with ΜΦ-specific TPE-FLIM parameters and the corresponding TPE-FLIM images of suspected ΜΦs were recorded. To prove the measured cells are ΜΦs, the skin biopsies were labeled by irradiating a squared area of 28×28 µm^2^ located near the suspected ΜΦs with a Ti:sapphire laser (Mai Tai XF, Spectra Physics, USA, 100 fs pulses at a repetition rate of 80 MHz) at a maximal power of 50 mW at 760 nm for 3 s. All incubations were performed at room temperature unless otherwise stated. In brief, sections were fixed for 10 min in cold acetone (–20°C) and rinsed in TBS (Agilent Technologies, Santa Clara, CA). For staining of ΜΦs, the ΜΦ-specific anti-CD68 (clone ab955) (Abcam, Cambridge, UK), Recombinant Anti-CD163 antibody [EPR14643-36] (clone ab189915) (Abcam) were used to account for M1 and M2 ΜΦ phenotypes, respectively. Slides were rinsed three times with TBS, and endogenous peroxidase was blocked with 3% H_2_O_2_ in TBS for 5 min followed by incubation with anti-mouse EnVision+ labeled polymer (Agilent Technologies) for 30 min. Slides were rinsed in TBS as before and incubated with AEC substrate-chromogen (Agilent Technologies) for 10 min. Nuclei were counterstained with Mayer’s hemalum solution (Merck, Darmstadt, Germany). Stained ΜΦs have a brown-red color, which enables to visually distinguish them from other cells and the ECM. After the staining procedure, target ΜΦs and squared labels of the skin sections were identified by light microscopy and overlaid with TPE-FLIM images matching an appropriate magnification and image orientations.

Specifically, CD68-stained M1 ΜΦs were counted in the papillary dermis region in each biopsy, and an average of 209±25 cells/mm² for the papillary dermis and an average of 140±76 cells/mm² for the reticular dermis for a 10 µm deep cryo-section was observed (Figure 1c). The density of the CD163 stained M2 ΜΦs was an average of 242±126 cells/mm² for the papillary dermis and an average of 107±60 cells/mm² for the reticular dermis for a 10 µm deep cryo-section (Figure 1d).

The ΜΦs search algorithm we then used was similar to that recently presented by our group for the identification of resting and activated mast cells in the papillary dermis (Kröger et al., 2020) and included the following steps: first, the papillary dermis (≈60–100 µm depth for volar forearm) was explored for fluorescent spots of 10–15 µm in size with irregular shape and a membrane extension having bright spots of about 1–3 µm. The TPE-FLIM parameters of the suspected bright areas were measured and matched those of M1 and M2 ΜΦs obtained in vitro and ex vivo.

To prove that the TPE-FLIM parameters of other dermal cells, which have detectable TPE-AF intensity, namely, mast cells and dendritic cells do not match or superimpose with TPE-FLIM parameters of ΜΦs, negative control measurements were performed. The procedure was similar as described for the verification of ΜΦs in skin biopsies using specific immunofluorescence, but six human skin cryo-sections were stained for the presence of mast cells and two for dendritic cells.

Staining of mast cells was done by blocking with serum-free protein followed by incubation for 1 hr with anti-tryptase antibody (clone AA1) diluted 1:1000 in antibody diluent (all Agilent Technologies). For staining of dendritic cells, anti-CD11c antibody (clone B-Ly6) (BD Biosciences, Franklin Lakes, NJ) was used after fixing the cryo-section for 10 min in cold acetone (–20°C) and rinsing with TBS.

### Statistical analysis and classification algorithm

Matlab R2016a (MathWorks, Natick, MA) was applied for descriptive statistics of all TPE-FLIM data. All results are indicated as mean ± standard deviation. Differences between distributions were compared using the nonparametric Kolmogorov-Smirnov test with a significance level of *α=*0.05. The decision tree classifier was modelled using Scikit-learn 0.22 in a Python 3.7 environment (Python Software Foundation, Wilmington, DE). A randomised training set, consisting of 50% of the complete data set, was used for training and validating the test set 10,000 times. The true positive and true negative rates were calculated from the confusion matrix and describe the quality of the classification and indicate type I and type II errors. For the decision tree (Breiman et al., 1984), the TPE-FLIM parameters *τ*_1_, *τ*_2_, *τ*_m_, *τ*_1_/*τ*_2_, *a*_1_, *a*_2_, *a*_1_/*a*_2_, (*a*_1_*−a*_2_)/(*a*_1_+*a*_2_), TPE-AF intensity, cell shape, and decay curve were used for each cell measured in vitro, ex vivo, and in vivo and hyperparametrically optimized (Yang and Shami, 2020). The feature vector was constructed as follows: 8 (TPE-FLIM parameters obtained after bi-exponential approximation of the decay curve), in total, 8 values. The ΜΦ size was not included in the feature vector for the classification model, as ΜΦs in vivo could have slightly different dimensions from those measured in vitro (in cell cultures) and ex vivo (in biopsies), caused by obscuring effects of surrounding dermal tissue. Here, 1 represents circular and 0 noncircular shape. The lifetimes calculated from the bi-exponential decay model were averaged over the whole cell, and the fluorescence intensity was normalized by optical power and averaged the pixel of interest and the 48 neighbouring square pixel.

In total, 110 ΜΦs in vitro, 20 ΜΦs ex vivo, 70 ΜΦs in vivo (for M1/M2 ratio see Table 1), 59 mast cells in vitro, 17 mast cells ex vivo, 82 mast cells in vivo, 14 dendritic cells in vitro, 6 fibroblasts in vitro, and 21 neutrophils in vitro were used as input for the model (399 cells in total). Given data vectors from $x_{i}\in R^{n}$ , *i*=1,…, *l* and a label vector $y_{i}\in R^{l}$ , where a decision tree recursively separates the data into two classes with the mode m represented as Q. For each node a split $\theta =\left(j,t_{m}\right)$ decided with the feature j and the threshold $t_{m}$ . The node split the data into subsets $Q_{left}(\theta )$ and $Q_{right}(\theta )$.

$$Q_{left}\left(\theta \right)=\left(x,y\right)|x_{j}<=t_{m}$$

$$Q_{right}(\theta )=Q\backslash Q_{right}(\theta )$$

The impurity was calculated by the impurity function *H*() at the mode *m*

G(Q,θ)=nleftNmH(Qleft(θ))+nrightNmH(Qright(θ))

With the parameters for minimized impurities, the subsets were recourse until *N_m_*=1.

The return values of the classification were 0 for M1 ΜΦs, 1 for M2 ΜΦs, and 2 for other dermal cells, 0 for ΜΦs and 1 for other dermal cells for node *m* in the region *R_m_* and *N_m_* observation, the proportion of class *k* observations in node *m* is $p_{mk}=1/N_{m}\sum_{x_{i}\in R_{m}}I(y_{i}=k)$ .

ROC curves served as a tool to determine the diagnostic abilities of the method, where the true positive rate was plotted against the false positive rate of the respective outcomes for both the categorization of ΜΦs against other dermal cells and M1 ΜΦs and M2 ΜΦs against other dermal cells.

## Figures and Tables

**Appendix 1—figure 1.**
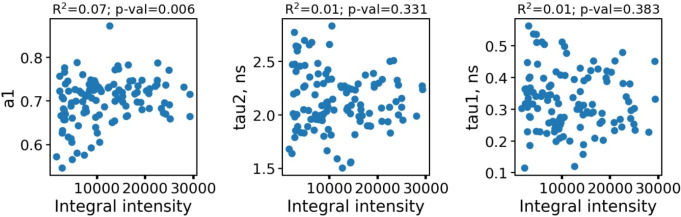
The dependence of fluorescence decay parameters for individual macrophages (*n*=110) on the integral fluorescence intensity (area under the fluorescence decay curve, upper row). As can be seen, the fluorescence decay parameters were independent on the intensity. To further confirm the absence of artefacts connected with parameters dependence on the FLIM data quality and processing algorithms, in Appendix 1—figure 2 the dependence of fluorescence decay curves parameters on χ^2^ is shown.

**Appendix 1—figure 2.**
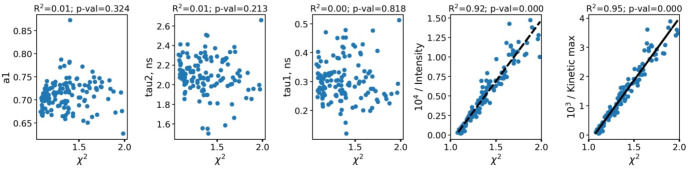
The dependence of fluorescence decay curves parameters on χ^2^. Each point corresponds to an individual macrophage cell (*n*=110). The dependence of χ^2^ on fluorescence intensity (both integral intensity per pixel and amplitude of the fluorescence decay curve, kinetic max) is a textbook knowledge—lower signal-to-noise ratio results in a worse fitting quality and higher χ^2^. Importantly, there was no correlation between the fluorescence decay parameters obtained from the decay curves and χ^2^; hence, there were no artifacts like lower fitting quality results in lower (or higher) values of fluorescence decay parameters. Summarizing, the fluorescence decay parameters obtained from bi-exponential fitting were independent on intensity (number of photons per pixel) and fitting quality, thus making it possible to use them as the descriptors for classification of cells. The comparison of fitting of the fluorescence decay for macrophages with two and three exponents ( Appendix 1—figure 3). As can be seen, an increase of the number of components does not result in an increase of the fitting quality.

**Appendix 1—figure 3.**
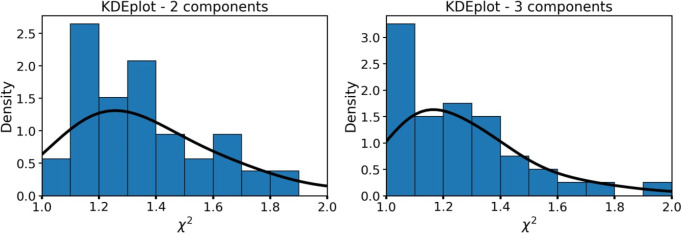
Distribution of the χ² for the bi-exponential (left) model and three-exponential (right) decay models.

## Data Availability

The data have been deposited in Dryad. The following dataset was generated: KrögerM DarvinM 2021Macrophage FLIM raw dataDryad Digital Repository10.5061/dryad.8gtht76q2

## Additional files

Transparent reporting form

## Appendix 1

### FLIM data analysis

Appendix 1—figure 1 shows the dependence of fluorescence decay parameters for macrophages on the integral fluorescence intensity.
